# Serum Renalase Levels Correlate with Disease Activity in Lupus Nephritis

**DOI:** 10.1371/journal.pone.0139627

**Published:** 2015-10-02

**Authors:** Chaojun Qi, Ling Wang, Minfang Zhang, Xinghua Shao, Xinbei Chang, Zhuping Fan, Qin Cao, Shan Mou, Qin Wang, Yucheng Yan, Gary Desir, Zhaohui Ni

**Affiliations:** 1 Department of Nephrology, Molecular Cell Lab for Kidney Disease, Renji Hospital, School of Medicine, Shanghai Jiao Tong University, Shanghai, China; 2 Health Care Center, Renji Hospital, School of Medicine, Shanghai Jiao Tong University, Shanghai, China; 3 Department of Medicine, Veterans Affairs Connecticut Healthcare System, Yale University, New Haven, Connecticut, United States of America; The University of Tokyo, JAPAN

## Abstract

**Introduction:**

Lupus nephritis (LN) is among the most serious complications of systemic lupus erythematosus (SLE), which causes significant morbidity and mortality. Renalase is a novel, kidney-secreted cytokine-like protein that promotes cell survival. Here, we aimed to investigate the relationship of serum renalase levels with LN and its role in the disease progression of LN.

**Methods:**

For this cross-sectional study, 67 LN patients and 35 healthy controls were enrolled. Seventeen active LN patients who received standard therapies were followed up for six months. Disease activity was determined by the SLE Disease Activity–2000 (SLEDAI-2K) scoring system and serum renalase amounts were determined by ELISA. Predictive value of renalase for disease activity was assessed. Furthermore, the expression of renalase in the kidneys of patients and macrophage infiltration was assessed by immunohistochemistry.

**Results:**

Serum renalase amounts were significantly higher in LN patients than in healthy controls. Moreover, patients with proliferative LN had more elevated serum renalase levels than Class V LN patients. In proliferative LN patients, serum renalase levels were significantly higher in patients with active LN than those with inactive LN. Serum renalase levels were positively correlated with SLEDAI-2K, 24-h urine protein excretion, ds-DNA and ESR but inversely correlated with serum albumin and C3. Renalase amounts decreased significantly after six-months of standard therapy. The performance of renalase as a marker for diagnosis of active LN was 0.906 with a cutoff value of 66.67 μg/ml. We also observed that the amount of renalase was significantly higher in glomerular of proliferative LN along with the co-expression of macrophages.

**Conclusion:**

Serum renalase levels were correlated with disease activity in LN. Serum renalase might serve as a potential indicator for disease activity in LN. The marked increase of glomerular renalase and its association with macrophages suggest that it might play an important role in disease progression of LN.

## Introduction

Lupus nephritis (LN) is one of the most serious complications of systemic lupus erythematosus (SLE), affecting 60% of patients and accounting for significant morbidity and mortality [1–4]. Diffuse proliferative glomerulonephritis is the most severe form of LN and the degree of proliferation may correlate with renal outcome [5]. Monocytes and macrophages are believed to play a critical role in the process of proliferation and fibrosis [6–9]. Although a number of immunosuppressive regimens had been tested for efficacy in the treatment of LN, 17% to 25% of individuals with LN will progress to end-stage renal disease (ESRD). It is widely believed that better renal outcome can be achieved only if methods for early and accurate diagnosis of LN are coupled with the prompt initiation of effective treatment protocols [10, 11].

Renalase is a monoamine oxidase which can be secreted into the blood from the kidney [12]. Previous studies found that renalase can regulate blood pressure by degrading catecholamines in the blood circulation [13]. Our recent studies indicated that renalase significantly alleviated renal injury when human proximal tubular (HK–2) cells were attacked by cisplatin and hydrogen peroxide. In acute kidney injury (AKI) mice model, administration of renalase is correlated with renal protection and less macrophage infiltration, whereas the renal injury and macrophage infiltration are more severe in renalase knock out mice model, which suggests the anti-inflammation role of renalase in kidney injury [14, 15]. The studies of organ transplant and serum renalase level found significantly up-regulation of renalase in kidney and heart recipients, which may also suggest the correlation between renalase and inflammation [16, 17]. Some interesting studies demonstrated that renalase was associated with type 1 diabetes, indicating it might be also involved in the development of autoimmune pancreatic destruction [18–20]. The finding of renalase associated with the development of autoimmune diabetes and organ transplant recipients suggests its role in the pathogenesis of diseases with a strong immune basis.

Despite the availability of data from the studies described above, the expression and clinical significance of renalase in LN patients remain unclear. In the present study, we sought to examine whether serum renalase levels were correlated with renal pathology and disease activity of LN and, in doing so, propose a possible role for renalase in this auto-immune and inflammation related disease.

## Materials and Methods

### Study population

The current cross-sectional study was approved by the ethics committee of Renji Hospital, School of Medicine, Shanghai Jiao Tong University, and all enrolled subjects signed written informed consent. LN patients were recruited from March 2012 to March 2013 from the inpatient department of the Renal Division of Renji Hospital. 157 patients with LN were screened and 67 subjects were selected. The exclusion criteria were as follows: life-threatening complications other than LN (e.g. heart failure, malignant tumor, infectious disease, central nervous system lupus), eGFR < 30ml/min/1.73 m^2^, pregnancy or age < 18.

The control group consisted of 35 healthy volunteers, paired by age and gender to the LN cohort. Healthy volunteers were enrolled from April 2012 to June 2012 when they did their medical examination in the Health Care Center, Renji Hospital. All of them were confirmed with no chronic diseases such as hypertension, diabetes and chronic kidney disease. They all gave informed consent for use of their serum samples.

Clinical histories and blood samples were collected at the study site. The presence of LN was established according to the American College of Rheumatology (ACR) revised criteria and all of the subjects were classified by renal biopsy [21]. Disease activity and renal disease activity were assessed by the SLE Disease Activity Index 2000 update (SLEDAI-2K) [22] and renal SLEDAI (rSLEDAI), respectively. The rSLEDAI (SLEDAI-2K renal scores) comprised haematuria, proteinuria, pyuria and urinary casts. LN patients were divided into two groups according to SLEDAI scores, active LN (SLEDAI ≥8) and inactive LN (SLEDAI <8).

Another 17 patients with active LN who received prednisone and immunosuppressive therapy were recruited and followed up for six months to evaluate the serum renalase levels before and after treatment. Of these, 12 received prednisone daily along with monthly infusions of cyclophosphamide (0.5-1mg/kg per day tapered after a few weeks combined with 500-1000mg/m^2^ cyclophosphamide every month) while 5 were prescribed prednisone and mycophenolate mofetil daily (0.5-1mg/kg per day tapered after a few weeks combined with mycophenolate mofetil 1.5-2g per day). Blood samples were collected before and after therapy.

### Laboratory parameters

Blood pressure was measured in the right arm after subjects were placed in the supine position for 5 minutes. Venous blood samples were then obtained from all subjects and the following parameters measured: complete blood count, serum creatinine (Scr), serum uric acid, blood urea nitrogen, lipids, complement C3 and C4, high-sensitivity C-reactive protein (hs-CRP), erythrocyte sedimentation rate (ESR). 24-hour urine and spot urine sample was collected to estimate urinary protein excretion. Anti-double-stranded DNA (dsDNA) antibody was detected by an enzyme-linked immunosorbent assay (ELISA, Euro Immune, China). All measurements were carried out by qualified technicians at the clinical laboratories of Renji Hospital. Estimated glomerular filtration rate (eGFR) was calculated using the modification of diet in renal disease (MDRD) formula {eGFR [ml/(min·1.73m^2^)] = 186 × (Scr, mg/dl)^-1.154^ × (age,year)^-0.203^ × 0.742(female) ×1.233}.

### Serum renalase determination

Blood samples were collected and centrifuged at 1000 × g for 10 minutes. The serum samples were collected and stored at -80°C until required for analysis. Serum renalase levels were measured using an ELISA kit for renalase according to the manufacturer’s protocol (USCN Life Science, WuHan, China)

### Evaluation of renal biopsies

Renal biopsies of LN patients enrolled in this study were obtained and evaluated using the International Society of Nephrology/Renal Pathology Society (ISN/RPS) classification [5]. Normal renal biopsy specimens were obtained from nephrectomy specimens of patients with suprarenal epitheliomas. In the current study, proliferative LN was defined as LN except ISN/RPS Class V which include Class III, IV, III+V, IV+V.

Frozen sections of LN renal biopsy were air dried, fixed with paraformaldehyde, and incubated overnight at 4°C with a mouse monoclonal antibody raised against the human macrophage marker, CD68 (ab49777, Abcam, Cambridge, MA) or a rabbit polyclonal antibody against the human endothelial cell marker, CD34 (14486-1-AP, Proteintech, USA) and a goat polyclonal antibody against human renalase (ab31291, Abcam, Cambridge, MA). The presence of either CD68 or CD34 and renalase was then assessed using a fluorescein isothiocyanate (FITC)-conjugated secondary anti-mouse antibody and a tetramethylrhodamine (TRITC)-conjugated secondary anti-goat antibody respectively. The nuclei were stained using 4',6-diamidino-2-phenylindole (DAPI, Vector Laboratories, Burlingame, CA). Fluorescent micrographs were generated using a fluorescence microscope (Nikon, Japan).

### THP–1 cell culture and analysis

THP–1 cells were cultured under standard conditions (e.g. humidified atmosphere of 5% CO_2_ at 37°C) in RPMI 1640 (Gibco, New York, USA) supplemented with 10% fetal bovine serum (Gibco, New York, USA), 50 IU/ml penicillin and 50 IU/ml streptomycin.THP–1 cells were stimulated with PMA (Sigma, St Louis, USA) for 72h at 100nM to obtain macrophages. The cultured macrophages were washed with PBS and thin-layer coated slides were prepared. Renalase and CD68 expression in these cells were determined using the same antibodies that were for the renal biopsies.

### Data analysis

Normally distributed data were presented as mean and standard deviations, while non-normally distributed variables were expressed as median and quartile. The Kolmogorov-Smirnov test was used to determine normal distribution. The Student’s T or Mann-Whitney U test was used to compare the variables between two groups when necessary. The differences before and after treatment were compared using the Wilcoxon singed-rank test. The comparison of all categorical variables such as frequency or percentage (%) was performed using either the Chi-square or Fisher’s exact tests. The correlation between two variables was determined by Pearson’s correlation coefficient analysis or Spearman’s rank correlation. The receiver operating characteristic (ROC) curve was performed by plotting sensitivity and specificity of serum renalase values according to Youden’s index. The multivariate logistic regression analysis was also used to determine the predicting factors for LN disease activity. The results were expressed as odds ratios (ORs) and their 95% CIs. A two-tailed P value < 0.05 was considered statistically significant.

## Results

### Serum renalase levels are higher in LN patients

67 LN patients and 35 healthy controls were enrolled in the current study. Of the LN patients, 5(7.5%), 35(52.2%), 7(10.5%), 12(17.9%) and 8(11.9%) had class III, class IV, class III+V, class IV+V and class V nephritis, respectively. The LN patients were comprised of 4 male and 63 female patients, with a mean age of 37.93±13.59 years. Healthy controls were confirmed with no chronic diseases, paired by age and gender to LN patients. Compared to the healthy controls, the level of serum renalase was significantly higher in LN patients (72.95±35.36 vs. 39.80±14.63 μg/ml, *P*<0.001). There were no significant differences in systolic and diastolic blood pressure between LN patients and healthy controls. Serum albumin, complement C3 and C4 levels were significantly lower in patients with LN than in health controls. Patients with LN also had higher ESR, dsDNA antibody levels compared to health controls (Table 1).

**Table 1:** Clinical characteristics of LN patients and healthy controls.

	Healthy controls(n = 35)	LN patients(n = 67)	P value
Male/female	2/33	4/63	0.786
Age	41.89±7.57	37.93±13.59	0.058
SBP (mmHg)	115.35±8.77	119.70±10.97	0.658
DBP (mmHg)	75.24±9.12	77.74±9.42	0.721
WBC (**×**10^9^)	5.53±1.44	6.38±3.24	0.070
Hemoglobin (g/L)	132.60±9.66	116.96±17.97	<0.001
Platelet (**×**10^9^)	217.74±55.85	192.91±66.62	0.062
Serum albumin (g/L)	44.46±2.60	33.59±7.98	<0.001
24hr urine protein (g/d)	0.0 (0.0–0.1)	1.38 (0.27–4.58)	<0.001
Serum creatinine (μmol/L)	58.70 (53.40–63.00)	62.00 (52.15–80.30)	0.433
eGFR (ml/min/1.73 m^2^)	107.63±18.76	105.59±35.91	0.755
Serum urea (mmol/L)	5.07±0.19	6.55±3.51	0.002
Serum uric acid(mmol/L)	272.43±51.98	345.85±106.29	<0.001
ESR(mm/h)	14.00 (8.00–19.00)	25.00 (9.00–57.50)	0.003
hsCRP(mg/L)	0.52 (0.22–1.08)	0.68 (0.17–2.54)	0.415
TG (mmol/L)	1.21±0.67	1.98±0.80	<0.001
TC (mmol/L)	4.73±0.85	5.80±1.97	<0.001
HDL (mmol/L)	1.64±0.40	1.58±0.60	0.573
LDL (mmol/L)	2.56±0.63	3.44±1.54	<0.001
ds-DNA (IU/L)	-	34.52±34.79	<0.001
C3 (mg/dl)	-	0.71±0.33	<0.001
C4 (mg/dl)	-	0.13±0.09	<0.001
SLEDAI	0	10.0 (4.0–16.0)	<0.001
Renalase (μg/ml)	39.80±14.63	72.95±35.36	<0.001

SBP, systolic blood pressure; DBP, diastolic blood pressure; WBC, white blood cell; eGFR, estimated glomerular filtration rate; ESR, erythrocyte sedimentation rate; hs-CRP, high sensitive C reaction protein; TG, total glycerin; TC, total cholesterol; HDL, high density lipoprotein; LDL, low density lipoprotein; ds-DNA, double strain DNA.

### Serum renalase levels are significantly elevated in proliferative LN

**Fig 1:**
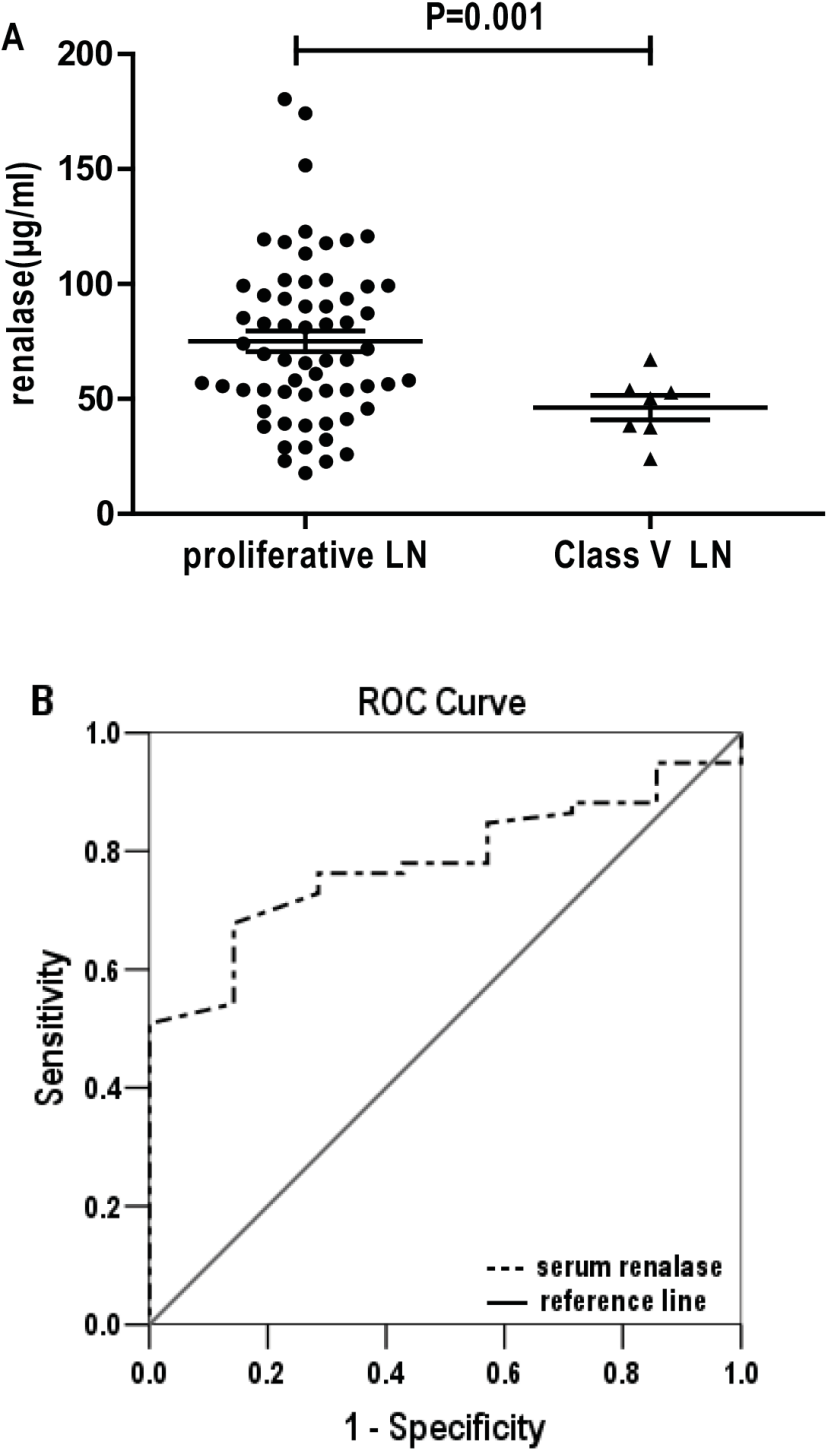
Serum renalase differentiates patients with proliferative LN from Class V LN. (A) Serum renalase levels were significantly higher in proliferative LN patients compared to Class V LN patients. Each dot represents the data of an individual. (B) Receiver operator characteristic curve (ROC) was performed to assess serum renalase as a predictive marker for proliferative LN. AUC was 0.780 while sensitivity and specificity were 67.80% and 85.71%, respectively, using a cutoff of 54.81 μg/ml.

According to the renal pathology, 67 LN patients were divided into two subsets, Class V and proliferative LN patients. The clinical manifestations of Class V and proliferative LN are listed in Table 2. Serum renalase levels in proliferative LN were found to be significantly higher compared to Class V LN patients (75.85±36.01 vs. 46.28±14.10 μg/ml, P = 0.001, Fig 1A). There were no significant differences in systolic, diastolic blood pressure, 24-hour urine protein excretion, serum albumin, creatinine, C3, C4, ESR, SLEDAI and rSLEDAI between Class V and proliferative LN. The anti-dsDNA antibody titers were significantly higher in proliferative LN patients than in Class V LN patients. The ROC curve to determine the diagnostic performance of serum renalase levels as a biomarker for pathological classification of LN was plotted. The area under the curve (AUC) of serum renalase to predict proliferative LN was 0.780 (95%CI, 0.652–0.907; P = 0.016). When the cutoff value was set at 54.81 μg/ml, the sensitivity for proliferative LN was 67.80% and the specificity was 85.71% (Fig 1B).

**Table 2:** Clinical manifestations of proliferative LN patients and Class V LN patients.

	Proliferative (n = 59)	Class V (n = 8)	P value
Age(years)	36.07±13.09	43.71±11.87	0.146
SBP(mmHg)	121.29±12.62	131.00±17.38	0.092
DBP(mmHg)	77.18±9.86	83.00±14.14	0.197
Ablumin (g/L)	33.57±8.33	33.68±5.10	0.963
24hr urine protein (g/d)	1.16 (0.26–4.50)	1.56 (0.84–3.56)	0.821
Creatinine (μmol/L)	54.1 (45.40–61.15)	62.1 (52.9±76.80)	0.552
Urea (mmol/L)	4.70 (4.15–6.90)	5.40 (4.10–7.85)	0.462
Uric acid(mmol/L)	347.25±105.51	335.45±118.92	0.771
eGFR (ml/min/1.73 m^2^)	103.31±34.55	121.85±43.54	0.174
ESR (mm/h)	32.76±29.18	34.63±24.48	0.864
C3 (mg/dl)	0.68±0.33	0.88±0.33	0.106
C4 (mg/dl)	0.12±0.09	0.18±0.06	0.114
ds-DNA (IU/L)	21.02 (9.52–65.38)	9.48 (4.90–12.30)	0.026
SLEDAI(score)	10(4–16)	6(4–11.5)	0.217
rSLEDAI(score)	8(0–12)	4(1–7)	0.362
Renalase (μg/ml)	75.85±36.01	46.28±14.10	0.001

SBP, systolic blood pressure; DBP, diastolic blood pressure; eGFR, estimated glomerular filtration rate; ESR, erythrocyte sedimentation rate; ds-DNA, double-strain DNA.

### Serum renalase levels correlate with disease activity in proliferative LN

**Fig 2:**
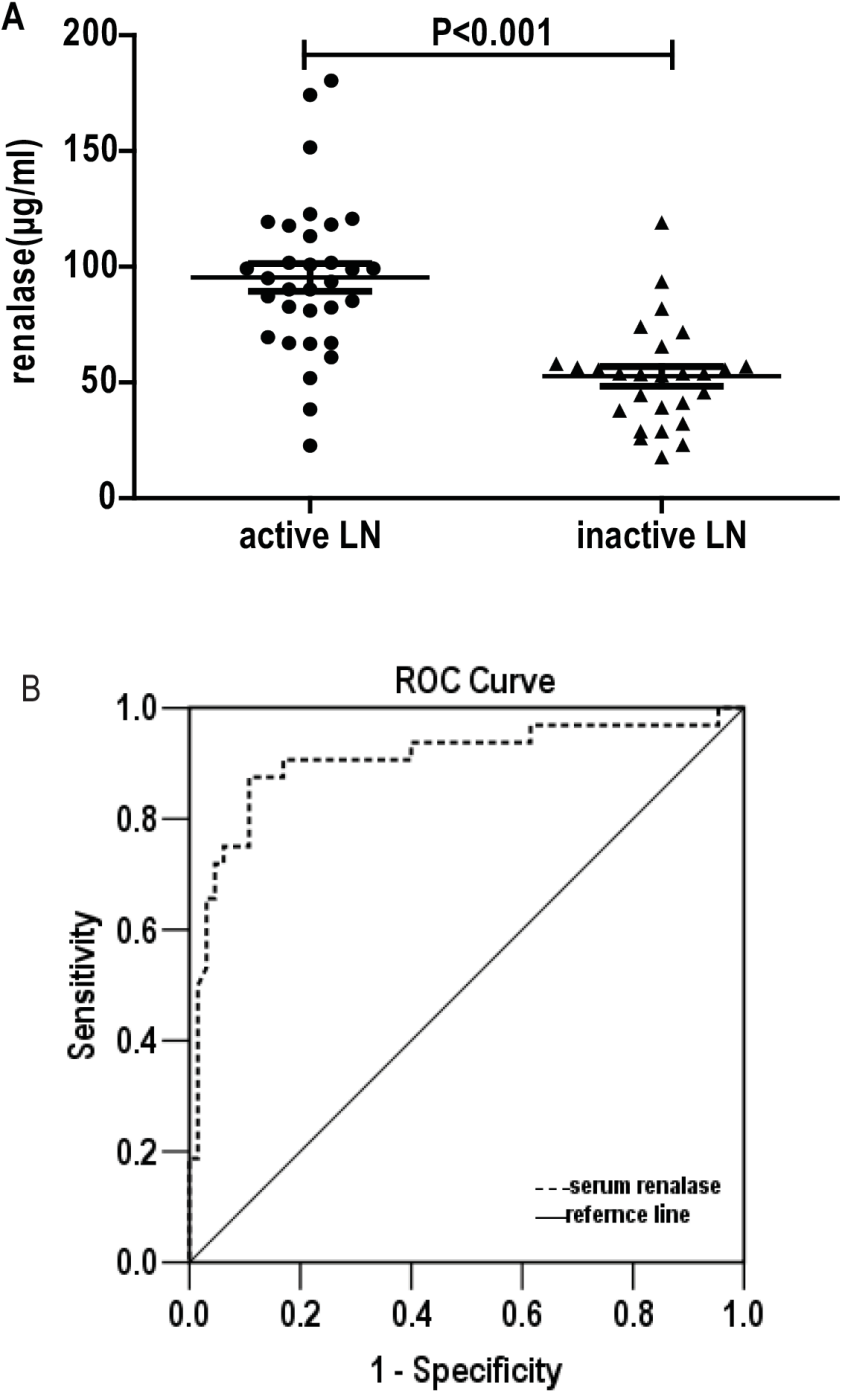
Serum renalase as a predictive marker for active LN. (A) The amount of serum renalase in subjects with active LN was significantly higher than in individuals with inactive LN. (B) Receiver operator characteristic curve (ROC) was performed to assess serum renalase as a predictive marker for active LN. The AUC was 0.906 using a cutoff value of 66.67 μg/ml while sensitivity was 87.5% and specificity was 89.2%.

To further investigate the correlation between serum renalase and disease activity in proliferative LN patients, 59 proliferative LN patients were divided into two groups according to their SLEDAI score. Patients with SLEDAI score ≥ 8 were considered as active LN. The clinical characteristics of the two groups are shown in Table 3. Serum renalase levels were significantly higher in patients with active LN compared to inactive LN (95.40±33.84 vs. 52.69±22.37 μg/ml, P<0.001, Fig 2A). Serum albumin and complement C3 and C4 levels were significantly lower in patients with active LN than in patients with inactive LN. Patients with active LN also had higher ESR and dsDNA antibody levels compared to those with inactive LN.

**Table 3:** Comparison of baseline characteristics in patients with active LN and inactive LN.

	Inactive LN(n = 27)	Active LN(n = 32)	P value
Male/female	0/27	1/31	0.774
Age	36.41±13.02	35.78±13.36	0.857
SBP (mmHg)	119.70±10.97	124.88±14.65	0.226
DBP (mmHg)	77.74±9.42	78.03±11.00	0.649
WBC (**×**10^9^)	6.54±2.57	5.85±3.55	0.404
Hemoglobin (g/L)	122.37±13.42	108.63±18.32	0.002
Platelet (**×**10^9^)	213.93±68.05	174.28±61.42	0.022
Serum albumin (g/L)	39.24±4.49	28.79±7.83	<0.001
24hr urine protein (g/d)	0.25(0.06–0.68)	3.31(1.20–6.42)	<0.001
Creatinine (μmol/L)	55.25 (49.78–63.70)	69.10(54.95–97.78)	0.004
eGFR (ml/min/1.73 m^2^)	117.90±29.86	91.06±33.86	0.003
Urea (mmol/L)	4.97±1.60	8.02±4.14	0.002
Uric acid(mmol/L)	301.1±82.54	386.12±108.20	0.001
ESR(mm/h)	10.00 (7.50–24.75)	43.00 (19.75–66.75)	<0.001
hsCRP(mg/L)	0.64 (0.18–1.94)	0.67 (0.17–3.22)	0.954
TG (mmol/L)	1.63±0.65	2.19±0.83	0.008
TC (mmol/L)	5.03±0.98	6.23±2.39	0.021
HDL (mmol/L)	1.63±0.44	1.44±0.65	0.214
LDL (mmol/L)	2.79±0.78	3.95±1.81	0.002
ds-DNA (IU/L)	17.24±19.22	55.48±37.16	<0.001
C3 (mg/dl)	0.88±0.20	0.52±0.33	<0.001
C4 (mg/dl)	0.16±0.07	0.10±0.09	0.007
SLEDAI(score)	4.0 (2.5–6.0)	16.0 (12.0–17.3)	<0.001
rSLEDAI(score)	0.0(0.0–4.0)	12.0(8.0–12.0)	<0.001
Renalase (μg/ml)	52.69±22.37	95.40±33.84	<0.001
Classification n (%)			0.179
Class III	2 (5.7%)	3 (8.6%)	
Class IV	14 (40.0%)	21 (60.0%)	
Class III+V	5 (17.1%)	1 (2.8%)	
Class IV+V	5 (14.3%)	7 (20.0%)	

SBP, systolic blood pressure; DBP, diastolic blood pressure; WBC, white blood cell; eGFR, estimated glomerular filtration rate; ESR, erythrocyte sedimentation rate; hs-CRP, high sensitive C reaction protein; TG, total glycerin; TC, total cholesterol; HDL, high density lipoprotein; LDL, low density lipoprotein; ds-DNA, double-strain DNA.

Univariate analysis of the data revealed positive correlations between serum renalase levels and SLEDAI (r^2^ = 0.32, P<0.001), ESR (r^2^ = 0.15, P = 0.003), and anti-dsDNA (r^2^ = 0.10, P = 0.013). The amount of serum renalase was negatively correlated with serum albumin (r^2^ = 0.25, P<0.001) and C3 levels (r^2^ = 0.17, P = 0.001). Neither systolic nor diastolic blood pressure was found to be correlated with serum renalase levels.

Serum renalase levels were also found to be positively related to 24-hour urine protein excretion (r^2^ = 0.417, P = 0.001) and rSLEDAI (r^2^ = 0.37, P<0.001).

The area under the ROC curve (Fig 2B) of serum renalase as a marker for active LN was 0.906 (95% CI: 0.830–0.981, P<0.001) with the cutoff point of 66.67 μg/ml, the sensitivity and specificity for distinguishing active and inactive LN were 87.5% and 89.2%, respectively. A step-wise multivariate logistic regression was used to verify the predicting factors for LN disease activity. Nine variables including age, gender, 24-hour urine excretion, serum albumin, complement C3, C4, serum creatinine, anti-dsDNA antibody titers, ESR and serum renalase were enrolled in the multivariate logistic regression. Only serum renalase and complement C3 showed statistical differences, indicating serum renalase (OR 0.928 and 95%CI 0.888–0.970, P = 0.001) and complement C3 (OR 44.715 and 95%CI 3.065–0.970, P = 0.005) could be independently indicators for LN disease activity.

### Effect of treatment on serum renalase levels

**Fig 3:**
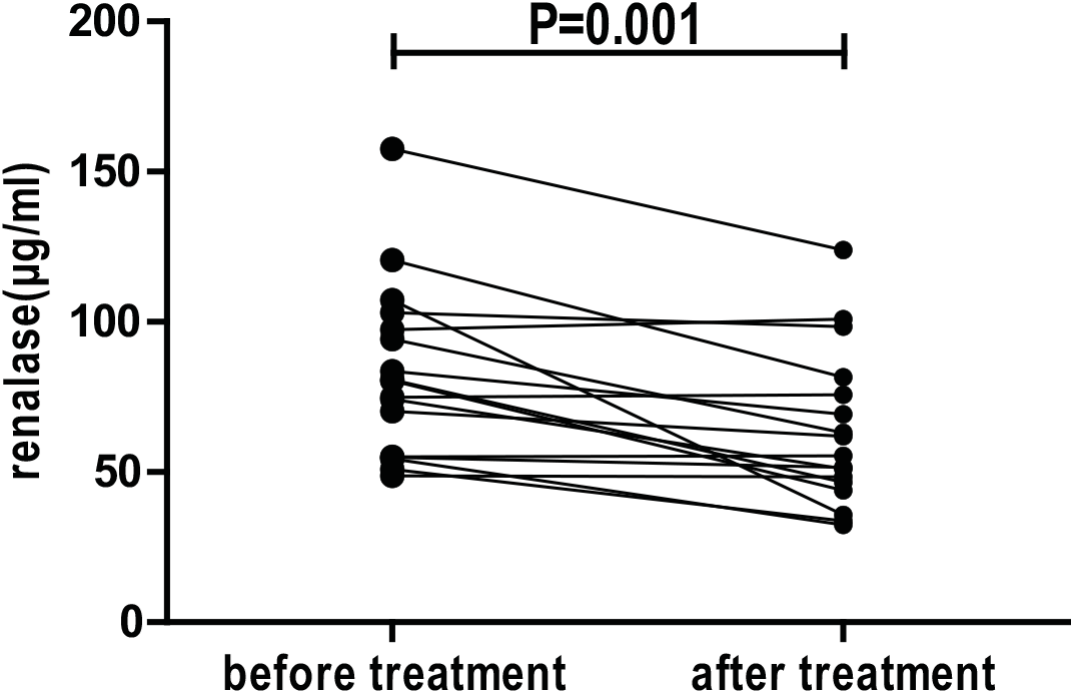
Comparison of serum renalase levels in patients with active LN before and after immunosuppressive treatment. Serum renalase levels decreased significantly after six-month immunosuppressive treatment.

To confirm the correlation between serum renalase and disease activity of LN, another 17 (2 with Class III, 10 with Class IV, 5 with Class IV+V) active LN patients were enrolled to compare serum renalase levels before and after treatment. They received a 6-month course of immunosuppressive therapy. Blood samples of LN patients were collected before and after treatment. Examination of the clinical parameters obtained before and after treatment (Table 4 **)** indicated a significant reduction in disease activity (e.g. ds-DNA level, ESR, proteinuria, SLEDAI scores, serum albumin, C3, C4, hemoglobin and WBC). And serum renalase levels were significantly decreased after treatment (82.84±28.72 vs. 63.16±25.81 μg/ml, P = 0.001 Fig 3).

**Table 4:** Comparison of clinical characteristics before and after treatment of 17 patients with active LN.

	Before treatment	After treatment	P value
WBC (×10^9^)	4.73±2.40	7.96±2.39	<0.001
Hemoglobin (g/L)	102.71±17.36	118.35±17.16	0.001
Platelet (×109)	144.71±56.92	226.41±47.13	<0.001
Serum albumin (g/L)	24.82±5.53	36.71±4.27	<0.001
24h urine protein (g/d)	7.20 (4.21–8.63)	0.43 (0.20–1.45)	<0.001
Creatinine (μmol/L)	68.20(55.70–91.80)	56.20(50.50–65.30)	0.005
eGFR(ml/min/1.73 m^2^)	97.49±27.90	117.67±34.00	0.025
Urea (mmol/L)	7.91±5.27	5.32±2.18	0.029
Uric acid (mmol/L)	441.75±128.21	364.71±111.84	0.046
ESR(mm/h)	40.00 (31.50–57.75)	14.50 (7.00–27.25)	<0.001
hsCRP (mg/L)	0.85 (0.30–1.77)	0.33 (0.16–1.06)	0.642
ds-DNA (IU/L)	58.56±36.50	15.78±16.07	0.005
C3 (mg/dl)	0.40±0.25	0.99±0.19	<0.001
C4 (mg/dl)	0.06±0.04	0.18±0.07	<0.001
SLEDAI(score)	16.0 (14.5–18.0)	6.0 (3.0–8.0)	<0.001
rSLEDAI(score)	12.0(8.0–12.0)	4.0(0.0–4.0)	0.001
Renalase (μg/ml)	82.84±28.72	63.16±25.81	0.001

WBC, white blood cell; eGFR, estimated glomerular filtration rate; ESR, erythrocyte edimentation rate; hs-CRP, high sensitive C reaction protein; ds-DNA, double-strain DNA.

### Glomerular renalase expression is upregulated in proliferative LN

**Fig 4:**
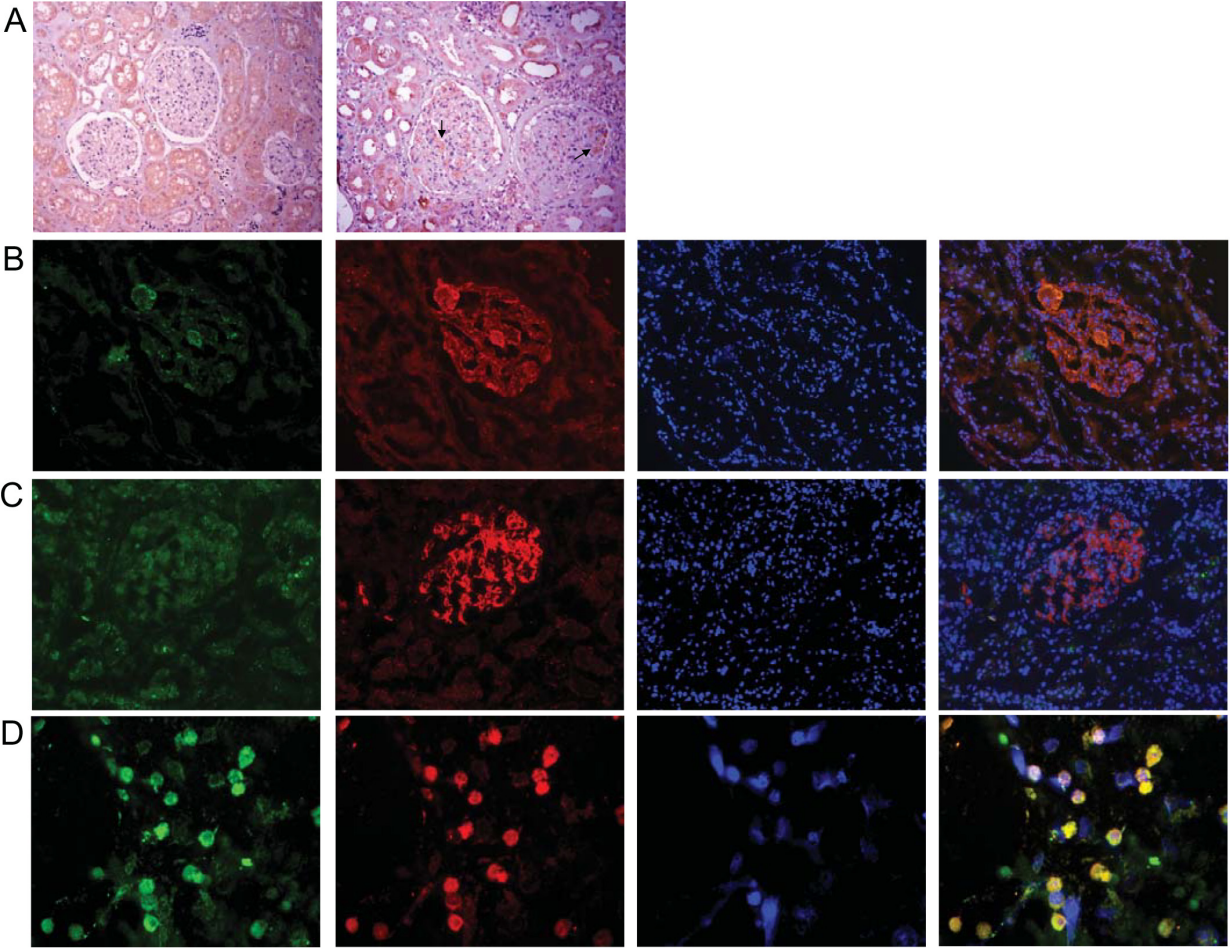
Expression of renalase in glomerular macrophages in active proliferative LN. (A) Left panel—kidney immunohistochemical staining for renalase of a control subject (20X); Right panel kidney immunohistochemical staining for renalase of an active LN patient (20X), arrows indicate representative renalase expression in glomeruli; (B) immunofluorescent staining of representative kidney sections of a patient with active LN, Left panel–staining with anti-CD68 antibody, Middle left- staining with anti-renalase antibody, Middle right -: nuclear staining, Right panel- merged picture; (C) immunofluorescent staining of representative kidney sections of a patient with active LN, Left panel—staining with anti-CD34 antibody, Middle left—staining with anti-renalase antibody, Middle right-: nuclear staining, Right panel—merged picture; (D) immunofluorescent staining of THP–1 cells in culture; Left panel—staining with anti-CD68 antibody, Middle left—staining with anti-renalase antibody, Middle right-: nuclear staining, Right panel—merged picture.

While renalase expression was undetectable in glomeruli of healthy control kidneys, it was markedly up-regulated in the glomeruli of proliferative LN subjects (Fig 4A). Compared with healthy controls, expression of the CD68 macrophage marker in glomeruli was increased in proliferative LN. Interestingly, renalase and CD68 expression were co-localized in glomeruli in proliferative LN (Fig 4B) suggesting that the increase in renalase expression might be a result of macrophage infiltration. Since renalase expression has also been detected in endothelial cells, co-staining with renalase and CD34 (an endothelial cell marker) was performed. As shown in Fig 4C, significant co-expression of renalase and CD34 was not detected in the glomeruli of proliferative LN. Renalase expression in macrophages was confirmed by staining the THP–1 cell line (Fig 4D).

## Discussion

SLE is a systemic autoimmune disease affecting a wide range of target organs and clinical manifestations with unpredictable flares and remissions [23, 24]. LN is one of the most serious complications of SLE since it is the major predictor of poor prognosis [23] and is considered as a classic example of immune complex induced injury resulting from the circulating antigen-antibody complexes and other mechanisms like *in situ* macrophage accumulation [25]. Biomarkers that are predictive for the development of active LN would be of significant value since early detection and treatment may positively affect renal outcome.

Complement C3 and C4, anti-dsDNA antibodies were currently used as markers for disease activity in LN. However, these autoimmune serologic markers are not sensitive and specific enough to be an optimal biomarker for LN activity. In our current study, we found that the level of renalase was significantly higher in LN patients compared to healthy controls, especially in proliferative LN patients. Moreover, in proliferative LN patients, patients with active LN had higher serum renalase levels compared to patients with inactive LN, indicating renalase might be correlated with disease activity in proliferative LN. Through correlation analysis, we confirmed the correlation between serum renalase and clinical activity of proliferative LN patients. The ROC curve also showed that serum renalase could be a good predictor for disease activity of proliferative LN. Besides, serum renalase levels decreased following immunosuppressive therapy along with the descending of anti-dsDNA antibodies, C3 and SLEDAI score. Furthermore, renalase expression was upregulated in the glomeruli of proliferative LN patients, suggesting that renalase expression and signaling may play a role in the pathogenesis of active LN.

Renalase was shown to be involved in the regulation of blood pressure and cardiovascular function [12, 26]. Although some other studies reported no association between serum renalase and blood pressure in hemodialysis or peritoneal dialysis patients [27, 28], the possibility of blood pressure influencing serum renalase may still exist. However, as there was no significant difference in blood pressure between groups, the influence of blood pressure may be minimal in our study.

Renal macrophages play an important role in cell proliferation and the development of kidney damage. Studies indicate that activated renal macrophages are markers of disease onset and poor outcome in LN [7, 29,30]. Studies in animal models of LN also suggest that the onset of proliferative glomerulonephritis and proteinuria is associated with endothelial activation, upregulation of inflammatory cytokines expression, and infiltration of macrophages that aggravate the inflammatory process [31]. In the present study, we found that renalase expression was not detected in the glomeruli under normal conditions. While patients with proliferative LN had significantly elevated serum renalase levels compared to those with Class V LN. Moreover, the expression of renalase was found markedly up-regulated in glomeruli of active proliferative LN and was co-localized with the macrophage in vivo and in vitro. The expression of renalase was also showed in macrophages of atherosclerotic plaque in Zhou’s research [32]. These findings suggest an important role of macrophages and in situ renalase in the disease progression of LN. Our previous data indicated that renalase could protect kidney from ischemic and toxic injury independent of its enzymatic function by anti-macrophage infiltration [14, 15]. We supposed that renalase may modulate macrophage function in a paracrine fashion, in which case, its signaling pathway may prove to be a novel therapeutic target for the treatment of LN.

There are some limitations in our study. The study was a single-center, cross-sectional study. A multi-center, prospective trial will be launched out to further understand and confirm the conclusions. The future study will be aimed at the time point when reanalase increases during LN, the differences of serum renalase levels in different types of kidney disease, whether renalase was involved in the pathogenesis of LN and its mechanism.

## Conclusions

Serum renalase levels were correlated with disease activity in LN patients making it a potential biomarker for future clinical studies. The marked increase of renalase and its association with macrophages in glomeruli suggested that it might play a role in pathogenesis of LN. Further studies are required to evaluate the utility of serum renalase as an indictor for disease activity in LN.
